# DNA analyses of a private collection of microbial green algae contribute to a better understanding of microbial diversity

**DOI:** 10.1186/1756-0500-7-592

**Published:** 2014-09-02

**Authors:** Ryo Hoshina

**Affiliations:** Department of Bioscience, Nagahama Institute of Bio-Science and Technology, Tamura 1266, 526-0829 Nagahama, Shiga, Japan

**Keywords:** Green algae, Internal transcribed spacer 2 (ITS2), Microbial diversity, Private collection, Species delimitation

## Abstract

**Background:**

DNA comparison is becoming the leading approach to the analysis of microbial diversity. For eukaryotes, the internal transcribed spacer 2 (ITS2) has emerged as a conspicuous molecule that is useful for distinguishing between species. Because of the small number of usable ITS data in GenBank, ITS2 sequence comparisons have only been used for limited taxa. However, major institutions with planktonic algal culture collections have now released small subunit (SSU) to ITS rDNA sequence data for their collections. This development has uplifted the level of molecular systematics for these algae.

**Results:**

Forty-three strains of green algae isolated from German inland waters were investigated by using SSU-ITS rDNA sequencing. The strains were isolated through the direct plating method. Many of the strains went extinct during the years of culture. Thus, it could be expected that the surviving strains would be common, vigorous species. Nevertheless, 12 strains did not match any known species for which rDNA sequences had been determined. Furthermore, the identity of one strain was uncertain even at the genus level.

**Conclusions:**

The aforementioned results show that long-forgotten and neglected collections may be of great significance in understanding microbial diversity, and that much work still needs to be done before the diversity of freshwater green algae can be fully described.

## Background

With the current rates of biodiversity loss, biodiversity conservation is receiving much attention. In many parts of the world, various animals and plants are being intensively studied, and conservation efforts for many species are well underway. Knowledge of microbial diversity, however, remains rudimentary. The current number of known microorganism species is a fraction of the diverse population of microorganisms [1, 2]. Much research, particularly the identification and description of species, is still required before microorganism diversity can be accurately quantified.

The development of the microscope made it possible to observe organisms smaller than dust; and with modern technological advances, the field of microbiology, especially microorganism systematics, has blossomed. DNA research methods have now facilitated the development of an entirely new level of systematics, thereby redefining the species concept. In particular, studies on various types of microorganisms have led to the development of their systematics. New techniques have revealed that many separate species were originally mistakenly grouped together as single morphospecies. A well-known example of this is *Chlorella*, which are common algae in natural water bodies. They are composed of simple, green, spherical cells containing a minimal set of organelles: one nucleus, one chloroplast, and one mitochondrion. These algae were originally considered one species, but small subunit (SSU) rDNA phylogenies have since shown that the group is in fact polyphyletic, consisting of morphospecies in more than one class [3, 4]. Based on the initial phylogenetic studies, Huss et al. [4] divided “true” *Chlorella* to four monophyletic species, including the type species *Chlorella vulgaris*. However, later studies identified some species that are morphologically different and draw their phylogenetic origins from the “true” *Chlorella* group e.g. [5–9].

The internal transcribed spacer 2 (ITS2) has emerged as a useful and conspicuous molecule for distinguishing between species. The ITS2 is located between 5.8S rRNA and LSU rRNA, which plays an important role in processing events during rRNA maturation e.g. [10, 11], and the secondary structures of ITS2 are highly conserved throughout the eukaryota [12]. The primary sequence of ITS2 region is highly conserved within species, but is highly divergent between species e.g. [13]. In general, the diversity of ITS2 sequence comparisons are either less than 2% or more than 10% (gaps are counted as a fifth character). This characteristic strongly encourages a species concept based on ITS2 sequence differences. Compensatory base changes (CBCs) in the ITS2 secondary structure also encourage species separation e.g. [14, 15]. The presence of at least one CBC has been correlated with the separation of two species, which is classified here by using the biological species concept based on the production of fertile offspring [14]. This hypothesis has been supported by data on various eukaryotic groups, including plants, fungi, and animals ([15] and references therein). Species concepts based on ITS2 differences in chlorophytes have gained wide acceptance. They also serve as a key to identifying and describing true microorganism diversity. This development also reveals the possible pitfalls of classification based on morphological characteristics.

In recent years, molecular phylogeny has been increasingly applied to the floristic study of planktonic green algae e.g. [16–19]. However, these studies analyzed SSU rDNA only and avoided species-level designation. This may have been largely because of the small amount of usable ITS data available in GenBank. However, major institutions holding algal culture collections, the Culture Collection of Algae and Protozoa (CCAP, UK), and the National Institute for Environmental Studies (NIES, Japan), have now released SSU to ITS rDNA sequence data for their collections. This has led to unprecedented developments in molecular systematics. Making use of these data, I have investigated the laboratory’s historical stocks of green algae, many of which have been neglected and forgotten about, which were isolated from German inland waters. Based on my findings, I discuss the potential of such archival lab stocks in supplementing our knowledge of biodiversity.

## Methods

### Cultures

Strains of green algae were established through simple methods. Water samples of several hundred microliters each were spread onto 1% agar plates containing 20% Gamborg’s B5 basal medium with mineral organics (Sigma-Aldrich, MO), to which was added 1/10 volume of lettuce juice medium [20] at pH 7.5. (The resulting medium is hereafter referred to as 1/5 GL medium.) The plates were incubated for two or three weeks under illumination by a light-emitting diode (LED) lamp (12 h/12 h light/dark cycle) at 15°C. Small single colonies (ca. 300 μm) that emerged were dissolved in water, and then spread onto a new plate under the same conditions. Single colonies that emerged from these cultures were transferred to liquid 1/5 GL medium (Table 1).

**Table 1:** **Strains sequenced in the study and their taxonomic designation**

Strain	rDNA sequence			Taxonomic designation
	Acc. no.	Coverage	SSU intron*	
GB1a	AB917097	SSU–ITS2		*Desmodesmus* sp.
GB1c	AB917098	SSU–ITS2		*‘Ankistrodesmus’ gracilis*
GB1d	AB917099	SSU–ITS2		*Pectinodesmus* sp.
GB1e	AB917100	SSU–ITS2		*Acutodesmus obliquus*
GB1g	AB917101	SSU–ITS2		*Acutodesmus obliquus*
GB1h	AB917102	SSU–ITS2		*Pectinodesmus* sp.
GB1j	AB917103	SSU–5′LSU	516	*Pectinodesmus* sp.
GB1k	AB917104, -105	SSU–5′LSU		*Micractinium* sp.
GS2i	AB917106	SSU–ITS2	516, 943, 1512	*Desmodesmus brasiliensis*
GS2j	AB917107	SSU–ITS2	516	*Desmodesmus opoliensis*
GS2k	AB917108	3′SSU–ITS2	—	*Desmodesmus opoliensis*
GS2L	AB917109	SSU–ITS2	516, 943, 1512	*Desmodesmus brasiliensis*
GS2m	AB917110	SSU–ITS2	516	*Desmodesmus opoliensis*
GS2n	AB917111	SSU–ITS2	516, 943, 1512	*Desmodesmus brasiliensis*
GS2o	AB917112	SSU–ITS2		*Desmodesmus armatus*
GS2p	AB917113	SSU–ITS2		*Desmodesmus armatus*
GS3a	AB917114	SSU–ITS2		*Acutodesmus obliquus*
GS3b	AB917115	3′SSU–5′LSU	—	*Acutodesmus obliquus*
GS3c	AB917116	SSU–ITS2	943	*Tetranephris brasiliensis*
GS3d	AB917117	3′SSU–5′LSU	—	*Acutodesmus obliquus*
GS3e	AB917118	SSU–ITS2		*Acutodesmus obliquus*
GS3f	AB917119	SSU–ITS2	943	*Tetranephris brasiliensis*
GS3g	AB917120	3′SSU–5′LSU	—	*Acutodesmus obliquus*
GS3h	AB917121	3′SSU–5′LSU	—	*Acutodesmus obliquus*
GS3i	AB917122	SSU–ITS2		*Acutodesmus obliquus*
GS3j	AB917123	3′SSU–ITS2	—	*Tetranephris brasiliensis*
GS3k	AB917124	3′SSU–5′LSU	—	*Acutodesmus obliquus*
GS3m	AB917125	SSU–ITS2		*Acutodesmus obliquus*
GS3n	AB917126	SSU–ITS2		*Acutodesmus obliquus*
GS3p	AB917127	SSU–ITS2		*Acutodesmus obliquus*
GM4a	AB917128	SSU–5′LSU		*Desmodesmus* sp.
GM4b	AB917129	SSU–5′LSU	156, 943, 1046, 1139, 1512	Selenastraceae sp.
GM4c	AB917130	SSU–ITS2	516, 1512	*Desmodesmus* sp.
GM4d	AB917131	SSU–5′LSU	516, 943, 1046	*Nephrochlamys subsolitaria*
GM4e	AB917132	SSU–5′LSU	40, 156, 516, 1046, 1139, 1512	*Neochloris* sp.
GM4f	AB917133	SSU–ITS2		*Desmodesmus armatus*
GM4g	AB917134	SSU–ITS2	516, 943, 1512	*Desmodesmus bicellularis*
GM4h	AB917135	SSU–ITS2	516, 943	*Desmodesmus armatus*
GM4i	AB917136	SSU–5′LSU	516	*Desmodesmus* sp.
GM4j	AB917137	SSU–5′LSU	516, 1512	*Desmodesmus* sp.
GM4k	AB917138	SSU–5′LSU		*Desmodesmus armatus*
GM4n	AB917139	SSU–5′LSU		*Desmodesmus pannonicus*
GA5a	AB917140	SSU–5′LSU		*Coccomyxa* sp.

*Insertion position corresponding to *Escherichia coli* rRNA gene.

Strain names indicate the water source in Germany from which the strain originated. GB1: a pond in the Botanischer Garten, Berlin, July 28, 2011; GS2: an artificial pond in the Mittlerer Schlossgarten, Stuttgart, July 31; GS3: a fountain in the Oberer Schlossgarten, Stuttgart, July 31; GM4: an artificial pond in Denninger Anger Park, Munich, Aug. 1; GA5: Alpsee Lake in the Ostallgäu district of Bavaria, Aug. 2.

The established colonies were cultured on two different media: first 1/5 GL, C medium [21], and then 0.1% Hyponex (Hyponex, Osaka). They were maintained under LED illumination (12 h/12 h light/dark cycle) at 10°C. The strains are available from the author upon request.

The culture stocks were observed under an Olympus BX60 light microscope (Olympus, Tokyo), and images were obtained with an Olympus DP72 digital camera.

### DNA extraction, amplification, and sequencing

DNA extractions using the DNeasy Plant Mini Kit (Qiagen, Düsseldorf, Germany) were performed. Polymerase chain reaction (PCR) was carried out to amplify SSU to ITS rDNA by using the primer pairs SR-1/SR-9 [22], SR-6 [22]/SR-12 k [23], INT-4 F [24]/ITS4 [25] and INT-4 F/HLR-3R [23]. PCR products were purified with NucleoSpin Gel and PCR Clean-up (Macherey-Nagel, Düren, Germany), and were directly sequenced by using the Operon DNA sequencing service (Operon Biotechnology, Tokyo). Some strains were amplified and sequenced only their ITS rDNA locus.

### Phylogenetic analyses

The SSU rDNA sequences were first checked for group I introns insertions (methods for group I intron ascertainment [26]). The joined exons were then submitted to the Basic Local Alignment Search Tool for Nucleotides (BLASTN, National Center for Biotechnology Information) for comparisons between sequences. Phylogenetic analyses of Chlorophyceae and Trebouxiophyceae were conducted separately. Taxon samplings for tree analyses were mainly based on recent papers [27–29]. The closest organisms for each strain that had been identified through BLASTN search were subsequently added. The SSU rDNA sequences were initially aligned automatically by using Clustal X2 software [30] and then aligned manually, taking into account secondary structure models for *C. vulgaris* [3] and *Heterochlorella luteoviridis* [31]. The 5′ and 3′ terminal regions were removed.

Two phylogenetic trees were constructed through the maximum likelihood (ML) method in PAUP 4.0b10 (Sinauer Associates, MA) or MEGA5 [32], and the neighbor-joining (NJ) method in the Saitou and Nei model in Clustal X2. The best-fit evolutionary models were identified by using Modeltest 3.7 [33] or MEGA5. By utilizing the results to derive the settings, a heuristic search using the NJ tree as the starting tree and a nearest-neighbor interchange swapping algorithm was performed. Bootstrap probabilities were computed for 100 (ML) and 1000 (NJ) replicates.

For advanced analyses at the genus or species level, phylogenetic analyses using both NJ and ML methods were conducted by examining ITS2 or SSU-ITS rDNA in conjunction with the results of recent studies of several different groups of species (*Scenedesmus*-related species: [34], *Desmodesmus*: [35], Chlorellaceae: [36]). ITS2 sequences were folded by using Mfold [37], and the secondary structures were used to aid manual alignment of those sequences and to check the presence/absence of CBC. For each tree, identical sequences were treated as one operational taxonomic unit.

## Results and Discussion

BLASTN analyses indicated that all strains analyzed in this study belonged to either Chlorophyceae or Trebouxiophyceae. These two classes were therefore treated separately.

### Chlorophyceans

**Figure 1:**
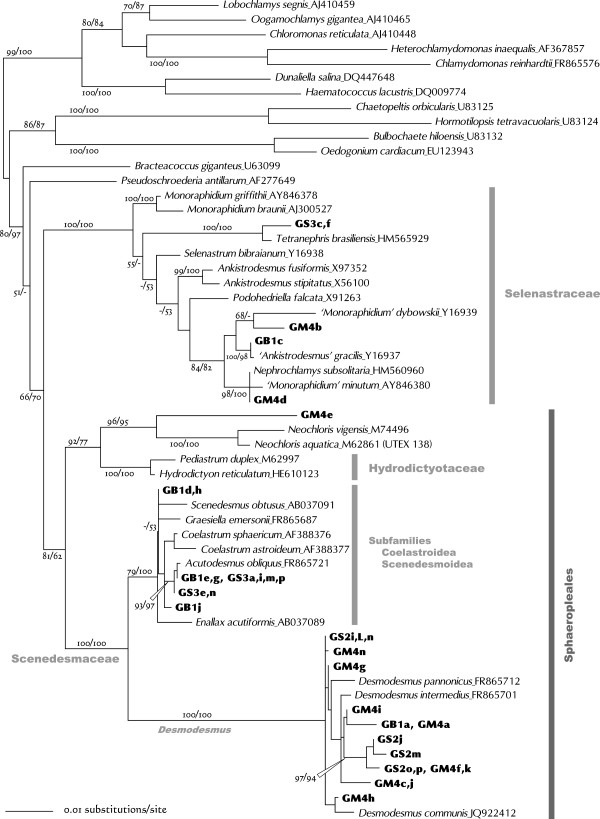
**Unrooted phylogenetic tree for Chlorophyceae based on SSU rDNA gene sequences (length 1605 nt).** Algal strains analyzed in this study were indicated by alphanumeric codes in bold. This tree was constructed by using the maximum likelihood (ML) method under the GTR + I + G evolutionary model. Numbers at each node represent bootstrap probabilities from ML/NJ analyses; only values above 50% are shown.

The SSU rDNA tree for members of Chlorophyceae separated them into three broad groups: Selenastraceae, the *Neochloris*-Hydrodictyotaceae clade, and Scenedesmaceae (Figure 1).

Five strains (GB1c, GS3c and -f, and GM4b and -d) were included in the Selenastraceae clade (Figure 1). Phylogenetic relationships for this group were constructed based on SSU rDNA analysis [28, 38, 39], which indicated that some genera were polyphyletic. However, many strains remained inadequately treated. Lacking data for more rapidly evolving molecules for this group, such as ITS, necessitated the use of the SSU rDNA phylogeny to determine the relationships between the cultured strains and previously analyzed members of this group.

**Figure 2:**
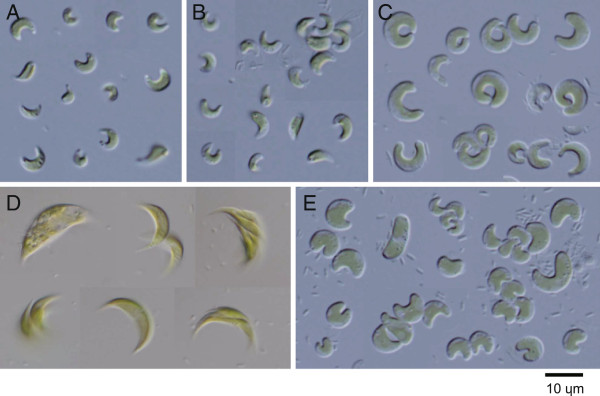
**Light microscopy images of algal strains identified as members of Selenastraceae from a private collection.** Strains were collected from German inland waters and were named according to their source (Table 1). **A**: GS3c, **B**: GS3f, **C**: GM4b, **D**: GB1c, **E**: GM4d.

GS3c and -f were grouped with *Tetranephris brasiliensis*, the type species for *Tetranephris*. There are three *Tetranephris* SSU rDNA sequences in GenBank (HM483517, HM565927, and HM565929), all of which are from *T. brasiliensis*. GS3c and -f are closest to the sequence HM565927, and have only one transition in 1601 aligned sites (data not shown). Light microscopy revealed solitary, crescent-shaped cells containing some granules (Figure 2A and B), which agrees with the morphological characteristic of *T. brasiliensis* and thus confirms that GS3c and -f are *T. brasiliensis* strains. Furthermore, the strain GS3j, which was analyzed by ITS sequencing only, had an ITS sequence and morphology identical to that of GS3c and -f (Table 1).

GM4b was grouped with *Monoraphidium dybowskii* in the ML tree (Figure 1), but this was not supported by the NJ analyses. Of note, however, was that the type species of *Monoraphidium*, *Monoraphidium griffithii*, was placed in a different group in this family. GM4b cells ranged from horseshoe- to donut-shaped (*M. dybowskii* cells are rhomboidal to labiate), with the diameters of the thickest parts between 2.5 and 4.0 μm (Figure 2C). Therefore, GM4b could not be identified even to genus level at this stage.

GB1c was clustered with *Ankistrodesmus gracilis* (Figure 1). Notably, the type species of *Ankistrodesmus*, *Ankistrodesmus fusiformis*, was placed in a different group in this family. GB1c cells were mainly crescent-shaped, 16–32 μm long, and 4–9 μm wide (Figure 2D). These morphocharacters are slightly different from that typical of *A. gracilis* (colony formation of 8–32 narrower crescent cells). As only one transition out of 1682 aligned sites between GB1c and *A. gracilis* was observed, GB1c was tentatively designated as a strain of *A. gracilis*.

GM4d was clustered with *Nephrochlamys subsolitaria* and *Monoraphidium minutum* (Figure 1). The GM4d sequence differed from that of *N. subsolitaria* by only two indels, and from that of *M. minutum* by five indels and four or five substitutions. The shape of GM4d cells resembled beans or curved cylinders (Figure 2E), similar to those of *N. subsolitaria* and *M. minutum*. Despite the pending taxonomic status of *M. minutum*, GM4d could be tentatively designated as a strain of *N. subsolitaria*.

**Figure 3:**
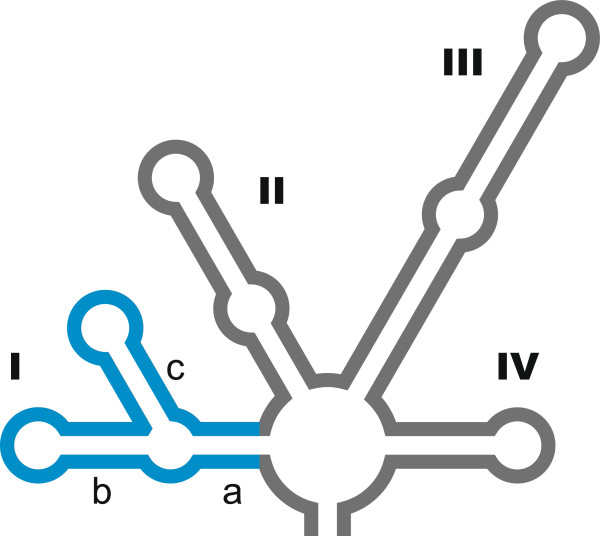
**ITS2 secondary structure that features a Y-shaped helix I (shown in blue).**

GM4e was grouped with the *Neochloris* species, and the *Pediastrum*-*Hydrodictyon* clade (Hydrodictyotaceae) was identified as a sister group to this clade (Figure 1). A close relationship between *Neochloris* and the Hydrodictyotaceae has been reported many times e.g. [40], and they have been classified as Sphaeropleales. This group includes Scenedesmaceae [41]. *Neochloris aquatica*, the type species of this genus, has been described based on UTEX 138 (the culture held at The Culture Collection of Algae, University of Texas) and on CCAP 254/5. However, their SSU rDNA sequences differ considerably from each other. When the ITS2 sequence of GM4e was submitted to BLASTN, it showed almost 90% similarity to the UTEX 138 strain of *N. aquatica*, AY577764, which includes 5.8S-ITS2 rDNA [42]. However, it was completely different from the CCAP 254/5 sequence (FR865697, including SSU-ITS2 rDNA). Preliminary analysis of the ITS2 secondary structure showed that both GM4e and *N. aquatica* UTEX 138 have a Y-shaped helix I (Figure 3), similar to that of Sphaeropleales [43–45]. When the CCAP 254/5 sequence was submitted to BLASTN, it showed more than 99% similarities (including ITSs) to several strains of *Chlorella vulgaris* (Trebouxiophyceae). It is possible, therefore, that the CCAP 254/5 strain had been confused with another strain.

**Figure 4:**
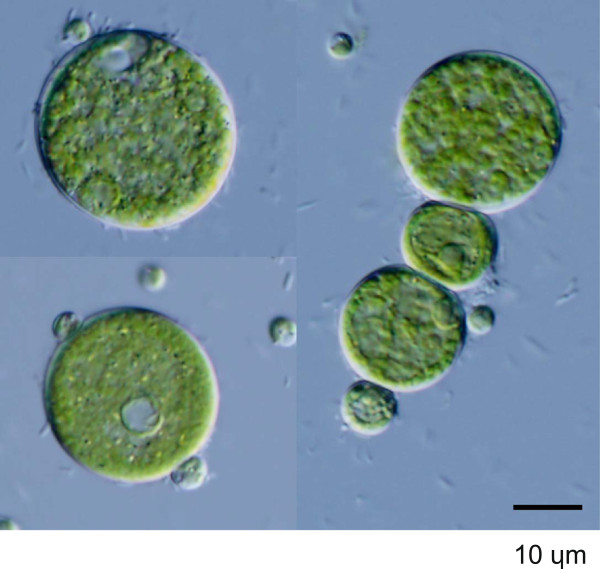
**Light microscopy image of cells from strain GM4e of a private collection.** This strain was collected from German inland waters and was named according to its source (Table 1).

Light microscopy revealed small to large spherical cells (3.2–32 μm) with a parietal chloroplast including one or more pronounced granules (Figure 4). Some cells contained segmentalized aggregations, which could be the development stage of zoospores. There is therefore no reason to exclude GM4e from *Neochloris*. AlgaeBase (http://www.algaebase.org) lists *Neochloris* as the taxonomically accepted genus name for eight species. Therefore, GM4e was designated as a species of *Neochloris*, although further comparative research with the other *Neochloris* species is required before this can be confirmed.

GM4e was particularly intron-rich; it contained six group I introns at S40, S156, S516, S1046, S1139, and S1512 (Table 1), which elongated its SSU rDNA to 4.3 kb. This ties with *Selenastrum capricornutum* [46] for the most intron insertions in the SSU rDNA. In contrast, previously described *Neochloris* species do not have any introns. Descriptions of these introns will be published elsewhere.

The remaining strains identified as Chrolophyceans all belonged to Scenedesmaceae, within which they were clearly separated into two clades, namely, Coelastroidea-Scenedesmoideae and *Desmodesmus* [47] (Figure 1).

**Figure 5:**
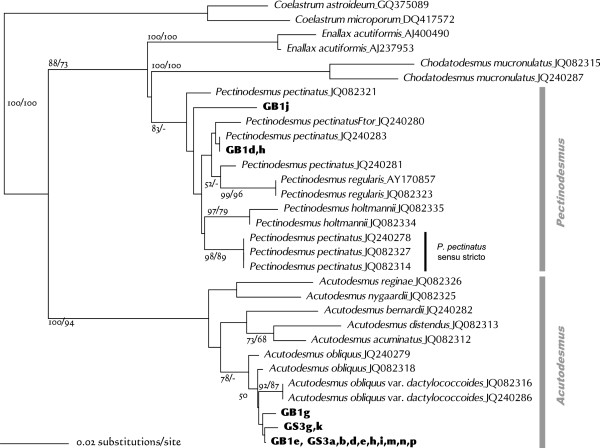
**Neighbor-joining tree inferred from ITS2 sequence comparisons (length 283 nt).** The *Coelastrum* species were used to root this tree. Numbers at each node represent bootstrap probabilities from NJ/ML (GTR + G model) analyses; only values above 50% are shown.

**Figure 6:**
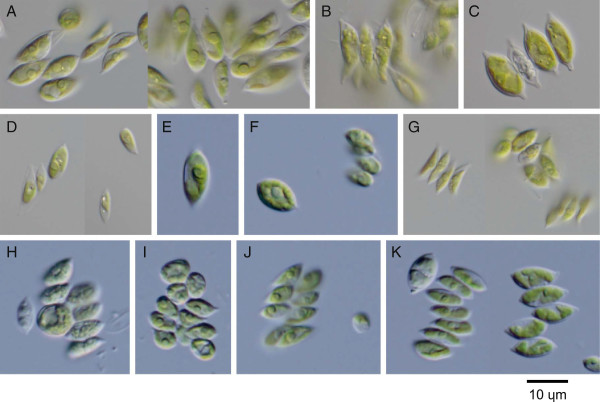
**Light microscope images of algal strains identified as members of** ***Scenedesmus*** **sensu lato.** Strains were collected from German inland waters and were named according to their source (Table 1). **A**: GB1j, **B**: GB1d, **C**: GB1h, **D**: GB1g, **E**: GS3g, **F**: GS3k, **G**: GB1e, **H**: GS3a, **I**: GS3b, **J**: GS3d, **K**: GS3h.

The *Scenedesmus*-related species were recently re-analyzed by using molecular phylogenetic techniques and electron microscopy e.g. [34, 47], which generated several new genera and many new species. Strains GB1j, -d, and -h were grouped with one of the new genera, *Pectinodesmus,* in the ITS2 analyses (Figure 5). All three strains formed four-cell coenobia comprising spindle cells (Figure 6A-C). This morphology coincides with that of *Pectinodesmus*, but it is insufficient for clear delineation of species in this genus [34]. *Pectinodesmus regularis* has been recognized as an independent species, but its genetic differences from other species are tenuous [34]. Furthermore, many genetically distinct strains dispersed throughout this clade are tentatively treated as *Pectinodesmus pectinatus* (Figure 5), obfuscating the distinction between species. Comparison of the ITS2 structures reveal that GB1j has one CBC and two hemi-CBCs (a compensatory base change on only one side of a pairing, the next-best “proof” of a CBC), and that GB1d and -h have one CBC when compared with *P. pectinatus* sensu stricto. Identification of strains GB1j, -d, and -h at species level must therefore be suspended until the phylogeny of the *Pectinodesmus* species becomes clear.

Thirteen strains (GB1e, -g, GS3a, -b, -d, -e, -g, -h, -i, -k, -m, -n, and -p) were clustered with known *Acutodesmus obliquus* strains (Figure 5). These culture strains had single- to eight-cell coenobia, and most cells were shorter and wider than those of typical *A. obliquus* strains (Figure 6D-K). This morphology could be related to the medium in which they were cultured. As CBC of these strains has not been found upon comparison with *A. obliquus* strains, they could be identified as *A. obliquus*.

Fawley et al. [16] suggested that a difference of even a single nucleotide in the SSU rDNA sequence could distinguish between species. However, this does not appear to be the case. Some strains with different SSU rDNA sequences had identical ITS sequences. For instance, there was one transition between the SSU rDNA sequences of GB1e and GS3e, but no substitutions in their ITS sequences.

**Figure 7:**
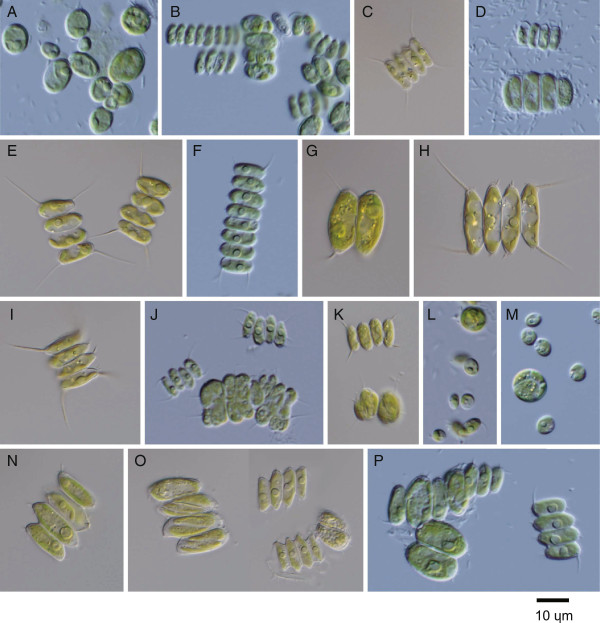
**Light microscope images of algal strains identified as** ***Desmodesmus*** **species from a private collection.** Strains were collected from German inland waters and were named according to their source (Table 1). **A**: GM4g, **B**: GM4i, **C**: GS2o, **D**: GM4h, **E**: GS2p, **F**: GM4k, **G**: GS2j, **H**: GS2k, **I**: GS2m, **J**: GM4a, **K**: GB1a, **L**: GM4c, **M**: GM4j, **N**: GS2i, **O**: GS2L, **P**: GM4n.

Seventeen strains were included in the *Desmodesmus* genus (Figure 1). Most of these strains had typical *Desmodesmus* forms, i.e., four- or eight-celled coenobia with or without spines on the terminal cells (Figure 7). Species of this genus have been characterized based on cell-shape, spines, and cell-wall appendages. However, several species could not be unambiguously distinguished even by scanning electron microscopy. As a result, recent studies have used molecular comparisons e.g. [48–50].

**Figure 8:**
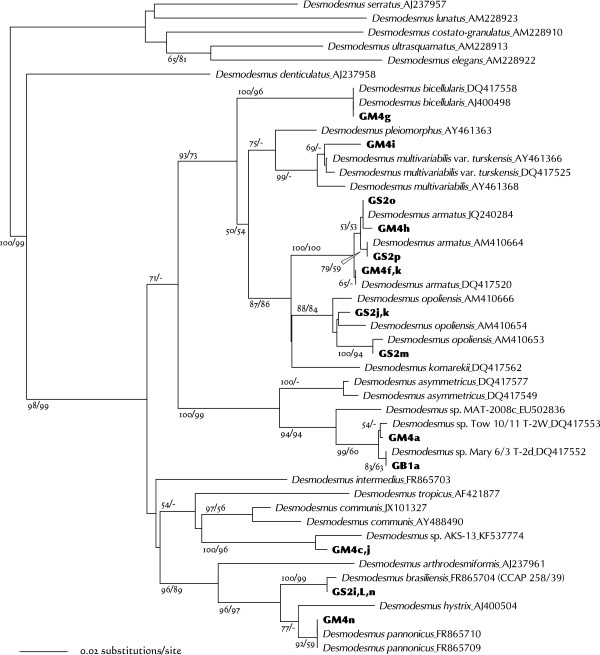
**Neighbor-joining tree inferred from ITS2 sequence comparisons (length, 276 nt).** *Desmodesmus serratus*, *D. lunatus*, *D. costato-granulatus*, *D. ultrasquamata*, and *D. elegans* were used to root this tree. Numbers at each node represent bootstrap probabilities from NJ/ML (SYM + I + G model) analyses; only values above 50% are shown.

Based on the ITS2 analyses, GM4g was clustered with *Desmodesmus bicellularis* strains (Figure 8). However, GM4g comprised single ellipsoids 5.1–15 μm long without any spines (Figure 7A), whereas *D. bicellularis* formed two- to eight-celled coenobia. Furthermore, Johnson et al. [48] observed irregularly shaped *D. bicellularis* cells with spine-like appendages (DQ417558). Because the GM4g ITS2 sequence was 100% identical to that of *D. bicellularis* strains, GM4g is probably a different morphological form of *D. bicellularis*.

GM4i formed four- or eight-cell coenobia. Although this morphology is not clearly shown in Figure 7B, each cell had a few small spines at the longitudinal ends. GM4i was clustered with *Desmodesmus multivariabilis* strains (Figure 8); however, it had a CBC at helix Ib when compared with these strains. Species-level identification is therefore not yet possible.

The strains GS2o, -p, GM4f, -h and -k were clustered with *Desmodesmus armatus* strains (Figure 8). They formed four- or eight-cell coenobia with long spines at the apices of the terminal cells, but the spines of GM4h were less obvious (Figure 7C-F). Based on the ITS2 phylogeny and the absence of CBC between these five and the previously identified *D. armatus* strains, they could be designated as *D. armatus* strains.

GS2j, -k, and -m were clustered with *Desmodesmus opoliensis* strains (Figure 8). In this study, the three *D. opoliensis* strains were employed from GenBank. Although these *D. opoliensis* ITS2 discrepancies are minor (5–7 changes out of 253 aligned sites), there is a CBC at helix IV and hemi-CBCs at helices Ic, II, and IV among them. Each of the variable sites found in the GS2j, -k, and -m strains applies to any of such variation. Their morphologies (two- or four-cell coenobia with long spines at the apices of terminal cells; Figure 7G-I) match those of the previously identified *D. opoliensis* strains. Therefore, they were tentatively identified as *D. opoliensis* strains.

GB1a and GM4a were placed in a clade comprising as-yet unidentified species, which is somewhat independent from previously described species of a sister clade to *Desmodesmus asymmetricus* (Figure 8). There was no CBC between GB1a, GM4a, and the two nameless strains Tow 10/11 T-2 W and Mary 6/3 T-2d, which were collected from Minnesota, USA [16]. They were all clearly separated from MAT-2008c by two CBCs and two hemi-CBCs. GB1a and GM4a formed two-, four- or eight-celled coenobia, of which each cell had a few spines (Figure 7J and K). Cells were comparatively small (3–6 × 8–13 μm).

Based on the ITS2 sequences, GM4c and -j were separated from the other *Desmodesmus* species. Tree analyses showed that their closest taxon was *Desmodesmus* sp. AKS-13 (Figure 8), but they were distinguished from each other by two hemi-CBCs at helices II and III. GM4c and -j cells were nearly spherical (3.5–10.5 μm), but some were ellipsoidal (up to 5.5 × 8 μm; Figure 7L and M).

GS2i, -L, and -n formed two- or four-cell coenobia (Figure 7N and O). Each cell had a few short spines, and some cells had granulated protoplasm. They were clustered with *Desmodesmus brasiliensis* CCAP 258/39 (Figure 8), with which there was no variation except for an N residue in the CCAP 258/39 sequence. CCAP has released five sequences (all including ITS2) for *D. brasiliensis* cultures. The ITS2 sequences of GS2i, -L, and -n closely matched the CCAP 258/39 sequence, but did not match the other four strains as closely (CCAP 258/40, 258/42, 258/43, and 258/44; these sequences were not used in this study). *D. brasiliensis* therefore contains several genetically distinct species. Because the authentic strain of *D. brasiliensis* has not yet been determined, I tentatively designate these three as strains of *D. brasiliensis*.

GM4n formed two- or four-cell coenobia or occurred as single cells (Figure 7P). Each cell had some spines, which projected in various directions. The ITS2 sequence for this strain was completely consistent with those of the *D. pannonicus* strains (Figure 8), so I designate GM4n as a strain of this species.

### Trebouxiophyceans

Of the 43 strains of green algae that were analyzed in this study, only two were from Trebouxiophyceae.

**Figure 9:**
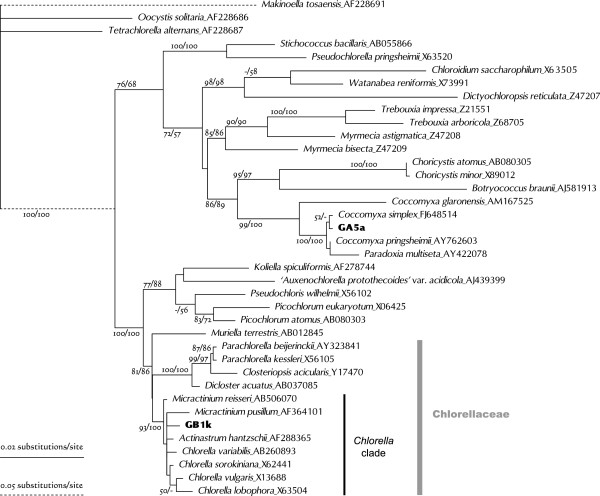
**Phylogenetic tree for Trebouxiophyceae inferred based on SSU rDNA gene sequences (length 1624 nt).** Algal strains analyzed in this study were indicated by alphanumeric codes in bold. This tree was rooted with *Makinoella*, *Oocystis*, and *Tetrachlorella* and was constructed by using the maximum likelihood (ML) method under the TN93 + I + G evolutionary model. Numbers at each node represent bootstrap probabilities from ML/NJ analyses; only values above 50% are shown.

**Figure 10:**
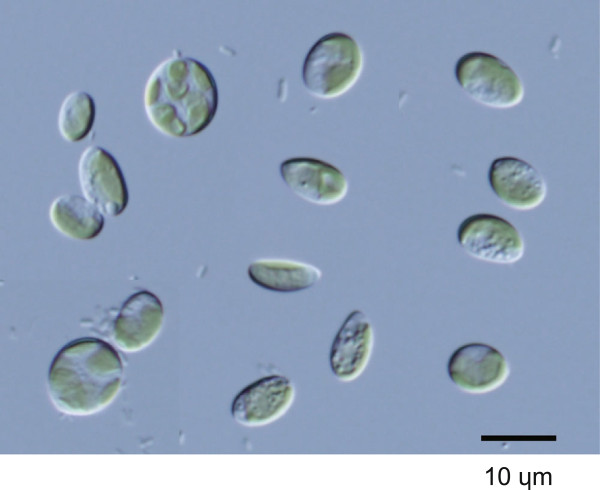
**Light microscopy image of the GA5a algal strain from a private collection.** This strain was collected from German inland waters and was named according to its source (Table 1).

**Figure 11:**
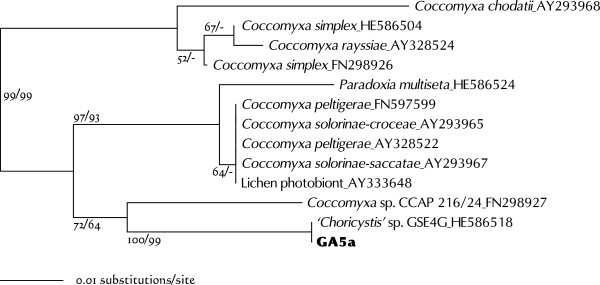
**Neighbor-joining tree inferred from ITS2 (helices I - III) sequence comparisons (length 194 nt).** Numbers at each node represent bootstrap probabilities from NJ/ML (GTR + I + G model) analyses; only values above 50% are shown.

Strain GA5a was clustered with *Coccomyxa* and *Paradoxia* species (Figure 9). These genera have been treated incertae sedis within the Trebouxiophyceae [51], and have not yet been revisited in detail by using molecular phylogenetic techniques. GA5a cells were simple ellipsoids, 7.0–11 μm long and 4.2–8.0 μm wide (Figure 10). They contained a girdle-shaped chloroplast without a visible pyrenoid. This morphology matches that of *Coccomyxa* or of the species classified hitherto as *Pseudococcomyxa* (shown as *Coccomyxa simplex* in Figures 9 and 11). BLASTN search for the GA5a ITS2 sequence found matches with several sequences. The GA5a sequence was identical to that of *Choricystis* sp. GSE4G (HE586518) (Figure 11). This was the case for both the ITS2 and the SSU-ITS rDNA. HE586518 was identified not as *Coccomyxa* or *Paradoxia* but as *Choricystis*. The morphology of *Paradoxia* was completely dissimilar to that of GA5a. In contrast, *Coccomyxa* and *Choricystis* have somewhat similar ellipsoidal single cells. However, *Choricystis* is a phylogenetically distinct genus, separate from *Coccomyxa* (Figure 9). Unfortunately, the authors who registered HE586518 have not yet published any information about this strain. For this reason, although GA5a is probably the same species as *Choricystis* sp. GSE4G, it may have to be designated as *Coccomyxa* sp. until publication of its further details.

**Figure 12:**
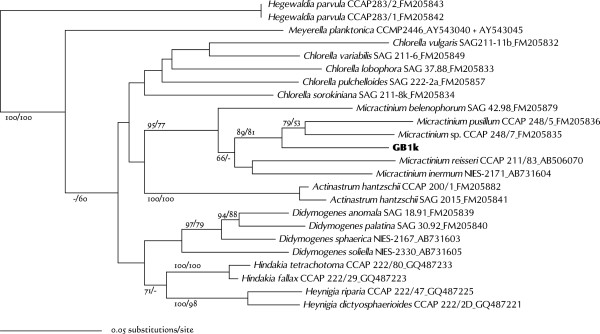
**Maximum likelihood tree constructed from SSU-ITS rDNA sequences (length 2487 nt).** *Hegewaldia parvula* was used to root this tree, which was constructed by using the GTR + I + G evolutionary model. Numbers at each node represent bootstrap probabilities from ML/NJ analyses; only values above 50% are shown.

**Figure 13:**
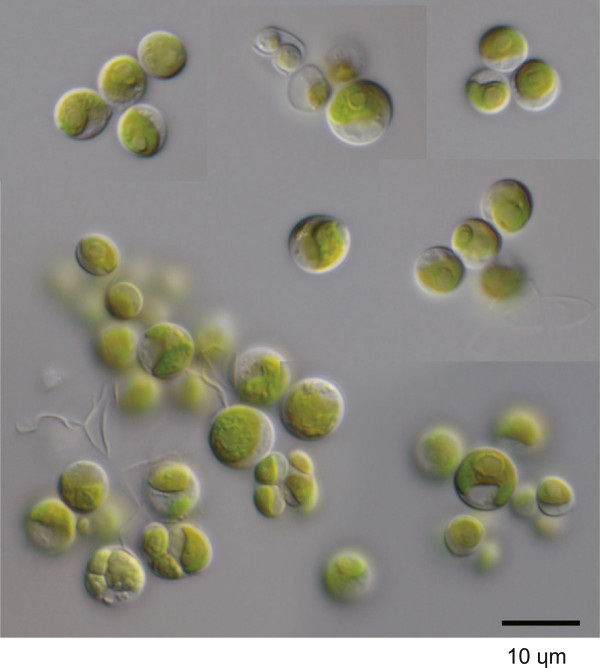
**Light microscopy image of the GB1k algal strain from a private collection.** This strain was collected from German inland waters and was named according to its source (Table 1).

**Figure 14:**
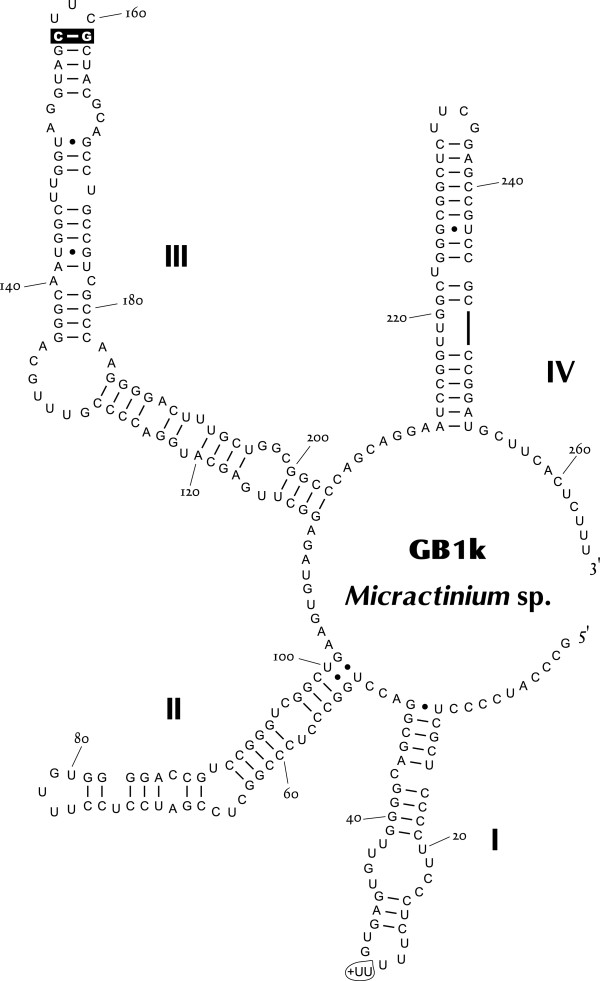
**Predicted ITS2 secondary structure diagram for GB1k.** The synapomorphic signature of genus *Micractinium*[56] is highlighted. The signature (+UU) at the terminal loop of helix I indicates the polymorphism (indels) in the GB1k genome.

Strain GB1k was included in the so-called “*Chlorella* clade” in Chlorellaceae (Figure 9). Members of this clade have been well studied, and recent studies have laid out guidelines for its genera and species delimitations [7, 36, 52–57]. GB1k was grouped with *Micractinium* in the SSU-ITS rDNA tree (Figure 12). This clade was independent of other clades, and had a long node and overall higher bootstraps. GB1k comprised spherical cells (4.5–8.5 μm) with a cup-shaped chloroplast containing a pyrenoid. Single cells were rare, and most cells formed coenobia (Figure 13). *Micractinium* is essentially a colonial species with several spines. However, recent studies have suggested that such morphological characteristics are unsuitable for defining genera within Chlorellaceae e.g. [52, 56]. In this regard, Luo et al. [56] instead examined the non-homoplasious synapomorphic molecular signature of the SSU rDNA or ITS2 sequence for the genus as well as its phylogenetic relationship. The only intelligible signature of *Micractinium* is C-G pairing at the tip of ITS2 helix III, which was found in GB1k (Figure 14). In ITS2 sequence comparisons, differences between GB1k and any other *Micractinium* species reached at least 25% (with gaps counted as fifth character). Although these large differences affect lengths or structures of helices and prevent accurate counting of CBC, there were at least two CBCs between GB1k and any other *Micractinium* species. Because the genus *Micractinium* is characterized by a colonial species with several spines, only two *Micractinium* species are described as having spherical cells (*Micractinium reisseri* and *Micractinium inermum*). GB1k is therefore probably a nondescript species of *Micractinium*.

## Conclusions

Many microbiology laboratories have vast microbial stocks isolated from natural environments. Some may have been collected as part of a search for new enzymes, antibiotics, or other applications. However, these stocks are often carelessly treated after the study has been completed, and are generally lost thereafter. My laboratory similarly owns large green algal stocks collected from natural water sources in various places. However, the number of culture stocks has been decreasing over time. There are numerous probable causes for this, including changes in the culture medium or other culture conditions, unexpected desiccation, and changes in culture management. If these stocks are likely to contain some interesting species, then it may be better to donate the strains to culture collection institutions, rather than to keep them for no reason. The present study focused on a private collection isolated from German inland waters, and examined their distinctiveness as a preliminary step toward donating the strains to a public collection. The method of establishing strains was very simple (q.v. Methods). This method may have selected only the species with the ability to grow faster and resistance to minor desiccation. Species that grow slowly or are unable to colonize an agar plate would be excluded from the selection. In addition, culture stocks have gone through a change in culture media, and some morphologically characteristic strains have already died out. That is, the culture stocks considered here are likely to be common and very vigorous species. Nevertheless, 12 strains out of 43 did not match any known species of which rDNA sequences have been determined (Table 1). One strain was nondescript even at the genus level. This fact suggests that the true diversity of freshwater green algae is still a long way from being fully described, and that even small laboratory stocks can contribute to our understanding of the diversity of microorganisms. I sincerely hope that this study prompts all researchers around the world to examine private microbial stocks and to publish their results.

There is intense debate about the diversity and distribution of eukaryotic microorganisms. One argument suggests that there is a limited number of species and that most of them are global in distribution e.g. [58]. The opposite argument states that large numbers of species exist although many species have possibly adapted locally; however, their simple morphology prevents discrimination between species e.g. [16, 59]. Now that microbes can be identified to species level by using DNA comparisons, as shown for some strains in this study, it is becoming apparent that a large number of freshwater green algal species exists in the world. Some strains in this study matched “brasiliensis” species (*Tetranephris*, *Desmodesmus*), and one (GB1a) matched a Fawley’s strain collected from the USA. There are therefore still many inconsistencies in patterns of species localization or ubiquity.

## Abbreviations

BLASTN: Basic local alignment search tool for nucleotides
CCAP: Culture collection of algae and protozoa
ITS: Internal transcribed spacer
NIES: National institute for environmental studies (Japan)
SAG: The culture collection of algae at Göttingen University
SSU: Small subunit
UTEX: The culture collection of algae, University of Texas.
